# [Expression of Concern] Novel insights into the role of HSP90 in cytoprotection of H_2_S against chemical hypoxia-induced injury in H9c2 cardiac myocytes

**DOI:** 10.3892/ijmm.2026.5880

**Published:** 2026-06-03

**Authors:** Zhanli Yang, Chuntao Yang, Liangcan Xiao, Xinxue Liao, Aiping Lan, Xiuyu Wang, Ruixian Guo, Peixi Chen, Chengheng Hu, Jianqiang Feng

Int J Mol Med 28: 397-403, 2011; DOI: 10.3892/ijmm.2011.682

Following the publication of this paper, and an Expression of Concern statement that was published to draw attention to the fact that the Editorial Office are in the process of consulting the authors regarding the fact that the photos shown in Fig. 5B and D were apparently matching images (doi: 10.3892/ijmm.2025.5618), we have been contacted again by another reader who has highlighted that Fig. 5C and F also contain an overlapping section, albeit that the image has been rotated through 90° and flipped vertically in panel (F).

We have contacted the authors again, asking them to provide an explanation for the apparent anomalies in the presentation of Fig. 5 in this paper, although up to this time, no response from them has been forthcoming. Owing to the fact that the Editorial Office has been made aware of these potential issues surrounding the scientific integrity of this paper, we are issuing a second Expression of Concern statement to notify readers of this potential problem while the Editorial Office continues to investigate this matter further.

